# Energy Intake and Satiety Responses of Eggs for Breakfast in Overweight and Obese Adults—A Crossover Study

**DOI:** 10.3390/ijerph17155583

**Published:** 2020-08-03

**Authors:** Jennifer B Keogh, Peter M Clifton

**Affiliations:** UniSA Clinical and Health Sciences, University of South Australia, Adelaide, SA 5000, Australia; peter.clifton@unisa.edu.au

**Keywords:** eggs, breakfast, energy intake, overweight, obesity

## Abstract

The type of food eaten for breakfast may determine the amount of food consumed at the next meal. This may be important when considering dietary advice for overweight and obese individuals who are trying to lose weight. The aim of the study was to investigate the energy intake and subjective sensations of hunger using a visual analogue scale (VAS) of a breakfast meal of eggs compared with a breakfast meal of cereal in overweight Australian adults. In a cross-over study, participants attended the University of South Australia’s Clinical Trial Facility on two separate days, one week apart. On each day participants consumed one of two isoenergetic breakfasts (1800 kJ), either eggs and toast or cereal with milk and orange juice. Fifty overweight or obese participants, 44 ± 21 years, 86 ± 14 kg, with a body mass index (BMI) of 31 ± 4 kg/m^2^ completed both study visits. Energy intake following the egg breakfast was significantly reduced compared with the cereal breakfast (4518 vs. 5283 kJ, *p* = 0.001). BMI and gender were unrelated to these effects. The sensation of hunger was less after the egg breakfast (*p* = 0.028 for diet by time interaction) and returned more quickly after the cereal breakfast. There were no effects of gender or age. Energy intake was reduced at an ad libitum lunch meal 4 hours after a breakfast meal containing eggs. The findings suggest that satiety responses of overweight and obese are not different to non-obese participants as our study confirms findings from studies conducted in different populations. Determining which foods may help overweight and obese individuals manage their food intake is important for diet planning.

## 1. Introduction

In 2017–2018, the prevalence of overweight and obesity in Australia was reported to be 67% in adults [1]. It would be helpful to understand the satiety effects of specific foods as overweight and obese individuals are frequently trying to lose weight or are advised to lose weight for the benefit of chronic health conditions. The Center for Disease Control in the Unites States reported that between 2013–2016, 49.1% of adults tried to lose weight in the previous 12 months with more women (56.4%) than men (41.7%) trying to lose weight [2].

Foods eaten at the first meal of the day, breakfast, may determine the energy intake at a subsequent meal [3]. Eggs contain high biological value protein which may satisfy hunger and improve satiety which may help reduce food intake later in the day [4]. 

In lean, young adults, egg breakfasts reduced postprandial glycaemic response and food intake at subsequent meal compared to a cereal [5]. Similarly in a study with lean men only, comparing eggs, with cereal and toast or a croissant there was increased satiety, less hunger and a lower desire to eat with a lower intake of energy at lunch and the evening meal after the egg breakfast [6]. In a study of normal or overweight men, participants ate less energy after an egg breakfast and ate fewer kilocalories in the 24-hour period [7]. They felt hungrier and less satisfied 3 h after the bagel breakfast [7]. In overweight and obese women, an egg breakfast produced greater feelings of satiety and reduced energy intake at lunch, for the remainder of the day and the next 36 h compared to an isocaloric bagel-based breakfast [8]. 

To date the studies conducted have been mainly in lean participants. Therefore, the aim of this study was to determine the energy intake and subjective acute satiety responses after a breakfast meal of two eggs compared with a cereal breakfast in overweight and obese men and women. The hypothesis was that the participants would have lower energy intake and increased satiety after the egg breakfast.

## 2. Materials and Methods 

This was a cross-over study comparing the effects of two breakfasts on subjective sensations of hunger and energy intake at a lunch meal eaten 4 h after breakfast. The order of the breakfasts was randomized. The sample size of 50 overweight or obese adults was based on previous satiety studies [5]. Participants were recruited form the Adelaide, South Australia community using print and social media and advertising flyers on the university campus. Participants were over 18 years, overweight with a body mass index (BMI) > 25 kg/m^2^ who self-reported that they were healthy, had no food allergies and were able to eat the study foods. Exclusion criteria were previous surgery for weight reduction, type 2 diabetes, systolic blood pressure > 160 mmHg, women who were or who wished to become pregnant and women who were breast feeding. Participants completed a Diet and Lifestyle Questionnaire to determine their eligibility to participate in the study. Questions related to weight, participation in other research at the time, pregnancy or breastfeeding, need for a special diet, food allergies or intolerances, surgery for weight reduction, diabetes, blood pressure, chronic medical medication, alcohol intake and smoking. Written informed consent was obtained from all participating volunteers. The study protocol was approved by the Ethics Committee of the University of South Australia.

### 2.1. Protocol

Participants attended the Clinical Trial Facility at the University of South Australia on 2 days, one week apart at 8–8.30 am. They were asked to fast after their evening meal for 12 h before their clinic visit with only water permitted and refrain from alcohol and strenuous exercise for 24 h before each visit. They were given instructions to eat the same evening meal at the same time before each visit. Height was measured once, weight was measured at both visits and BMI calculated. Following the protocol by Flint (2000), prior to eating breakfast participants were asked to complete a 100 mm visual analogue scale (VAS) and then eat a breakfast meal [6]. There were eight questions on the scale (Table 1) as follows: How hungry do you feel? How satisfied do you feel? How full do you feel? How much do you think you can eat? Would you like to eat something sweet? Would you like to eat something salty? Would you like to eat something savory? Would you like to eat something fatty? Participants were asked to mark the 100 mm scale. For the first question How hungry do you feel? the left-hand side of the scale read “I am not hungry at all” and the right-hand side read “I have never been more hungry”. The other seven questions followed the same pattern. Subsequent VAS responses were collected 15, 45, 75, 105, 135, 165, 195 and 225 min after finishing breakfast. The breakfast meals were 2 eggs with 2 slices of bread/toast and 10 g margarine (1800 kJ, 25 g protein, 23.5 g fat, 28 g carbohydrate, 7 g fibre) or a bran containing cereal with sugar, milk and orange juice (1788 kJ, 11 g protein, 5 g fat, 73 g carbohydrate, 11 g fibre). An ad libitum lunch was provided 4 h after breakfast. Only water was allowed after breakfast during the 4 h before lunch. Using a modified protocol from Bowen (2007) participants were offered large servings of pasta and tomato sauce (600 g of each) [9]. Foods were weighed to the nearest gram before and after lunch using digital scales. Energy and nutrient composition were calculated using Food Works 8.0 (Xyris Software, Highgate Hill, Australia). Participant support: Participants were provided with a AUD 100 voucher at the end of the study.

### 2.2. Statistics

Results are expressed as Mean ± SD unless otherwise stated. As this was a cross-over design analysis of variance (ANOVA) with repeated measures was used to determine the effect of time and treatment within subjects. Significance was set at *p* < 0.05. The VAS was analysed as given by Flint [10]. Baseline values were subtracted from postprandial responses to normalize between-subject differences. Statistical analysis was performed using SPSS 24.0 (IBM, New York, USA).

## 3. Results

A total of 53 participants commenced and 50 completed the study. Two participants did not attend for their first visit and one participant was unable to complete the second visit for personal reasons unrelated to the study. 

### 3.1. Baseline Characteristics 

Participants who completed the study were 44 ± 21 years, 86 ± 14 kg and BMI 31 ± 4 kg/m^2^. Weight and BMI were the same on both visits. Baseline characteristics for men and women are shown in Table 1.

### 3.2. Energy and Macronutrient Intake at Lunch

There was a significant difference in energy intake after the egg breakfast compared to after the cereal breakfast, 4518 ± 1593 vs. 5284 ± 1814), *p* = 0.001 (Table 2). BMI and gender were not related to this difference. Fat (*p* = 0.002), protein (*p* = 0.000) and carbohydrate intakes (*p* = 0.001) were statistically different after the breakfast study. There were no effects of gender, BMI, age or order of the breakfasts on any variable. Weight of food consumed at lunch was less after the egg breakfast (451 ± 184 g vs. 534 ± 193 g, *p* < 0.01).

### 3.3. Subjective Measures of Satiety 

#### 3.3.1. Hunger

**Figure 1:**
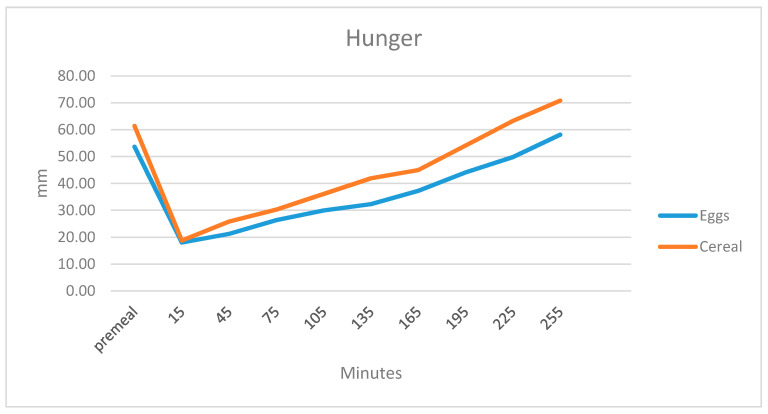
Subjective sensations of hunger before and 15, 45, 75, 105, 135, 165, 195 and 225 min after finishing breakfast on a 100-mm visual analogue scale (VAS).

Participants responded to the question “How hungry do you feel?” Overall, there was a difference in the subjective sensation of hunger between the breakfasts such that participants felt less hungry after the egg breakfast (Breakfast *p* = 0.006, Time *p* = 0.000, Diet by time interaction *p* = 0.028). There was no effect of gender and no effect of age. The addition of weight as a co-variate was significant, *p* = 0.014 and made the diet by time effect stronger, *p* = 0.004. The effect was similar for BMI, *p* = 0.021 for the interaction between diet and time and BMI and *p* = 0.008 for the diet by time interaction. Hunger returned to baseline more quickly in the cereal breakfast compared to the egg breakfast, *p* = 0.000, at the reading taken prior to lunch (Figure 1).

#### 3.3.2. Satisfaction

**Figure 2:**
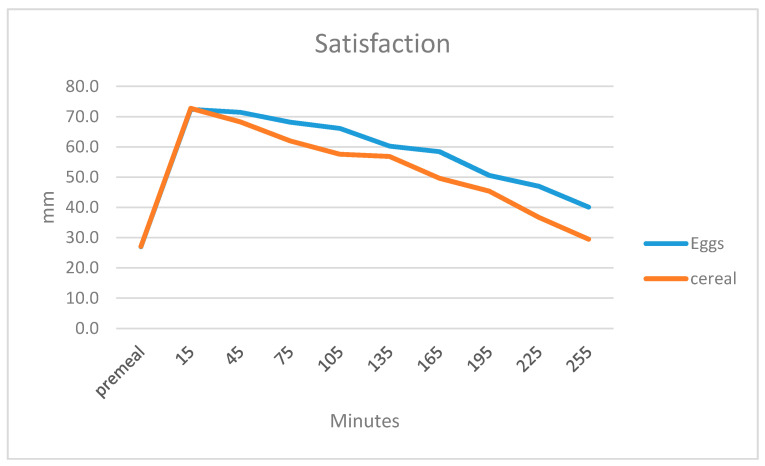
Subjective sensations of satisfaction before and 15, 45, 75, 105, 135, 165, 195 and 225 min after finishing breakfast on a 100-mm VAS scale.

Participants responded to the question “How satisfied do you feel?” Overall there was a small difference in the subjective sensation of feeling satisfied such that the participants felt more satisfied after the egg breakfast *p* = 0.05, the effect of time was significant, *p* = 0.000, with a trend towards a diet by time interaction, *p* = 0.072. The feeling of being satisfied fell more quickly after the cereal breakfast than after the egg breakfast (Figure 2).

#### 3.3.3. Fullness

**Figure 3:**
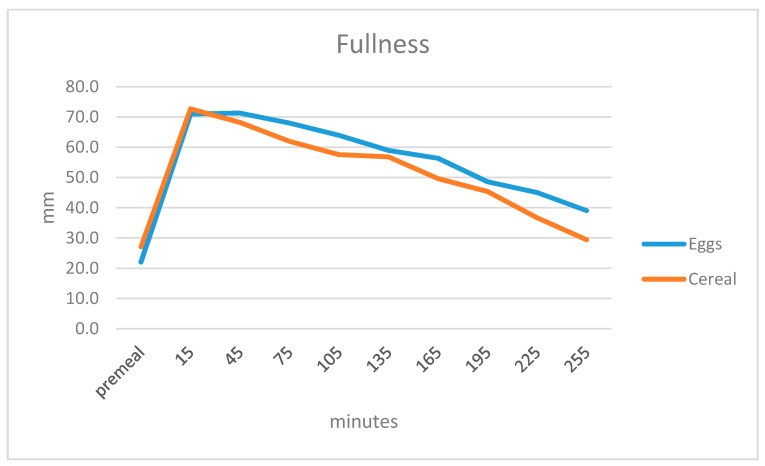
Subjective sensations of fullness before and 15, 45, 75, 105, 135, 165, 195 and 225 min after finishing breakfast on a 100-mm VAS scale.

Participants responded to the question “How full do you feel?” Overall, there was a small difference in the subjective feeling of fullness (*p* = 0.019) such that participants felt fuller after the egg breakfast. There was an effect of time (*p* = 0000) and a diet by time interaction (*p* = 0.015) (Figure 3).

#### 3.3.4. Desire to Eat

**Figure 4:**
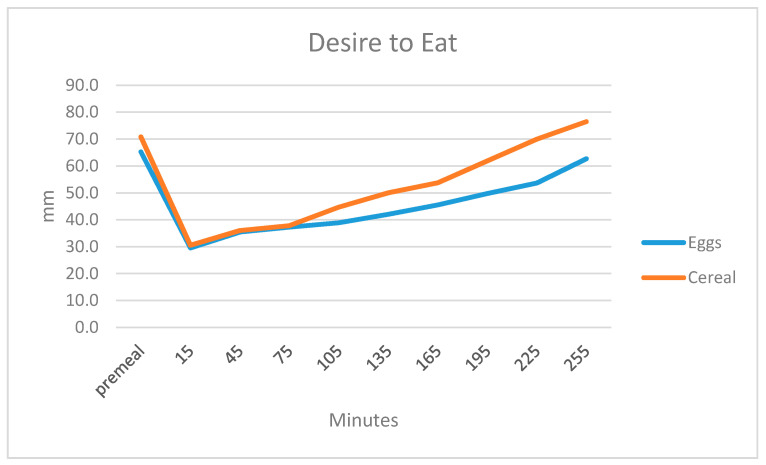
Desire to eat before and 15, 45, 75, 105, 135, 165, 195 and 225 min after finishing breakfast on a 100-mm VAS scale.

Participants responded to the question “How much do you think you can eat?” Participants thought they could eat less after the egg breakfast. There was an effect of time (*p* = 0.007) and a diet by time interaction (*p* = 0.000) (Figure 4).

#### 3.3.5. Desire to Eat Sweet Foods

Participants responded to the question “Would you like to eat something sweet?” Overall, there was an effect of time (*p* = 0.000) and a difference between the breakfasts such that overall after the egg breakfast participants desire for sweet foods was less than after the cereal breakfast (*p* = 0.049). However, the values had converged by the end of the morning.

#### 3.3.6. Desire to Eat Salty Foods

Participants responded to the question “Would you like to eat something salty?” There was a difference between the breakfasts (*p* = 0.044) with an effect of time (*p* = 0.000) and a breakfast X time interaction (*p* = 0.001) such that overall after the egg breakfast participants desire for salty food was greater than after the cereal breakfast.

#### 3.3.7. Desire to Eat Savoury Foods

Participants responded to the question “Would you like to eat something savoury?” There was no difference between the breakfasts. There was an effect of time (*p* = 0.000) with no breakfast by time interaction.

#### 3.3.8. Would You Like to Eat Fatty Foods

Participants responded to the question “Would you like to eat something fatty?” There was no difference between the breakfasts. There was an effect of time (*p* = 0.000) and time by meal interaction (*p* = 0.012) such that the desire to eat fatty food was greater in the cereal group.

## 4. Discussion

The main finding of this study was that eating eggs for breakfast resulted in a lower energy intake at an ad libitum lunch in this group of overweight and obese participants. Supporting this finding, there was a difference in the subjective sensation of hunger between the breakfasts such that participants felt less hungry after the egg breakfast. Participants felt more satisfied, felt fuller and thought they could eat less after the egg breakfast. This may be important given the prevalence of overweight and obesity and public health recommendations that individuals lose weight. In 2017–2018, the prevalence of overweight and obesity in Australia was reported to be 67% in adults [1]. Our study confirms findings from studies conducted in non-overweight/obese populations. Bonnema (2016) found that an egg breakfast meal produced greater overall satiety and reduced postprandial glycaemic response and food intake at subsequent meal compared to a cereal-based breakfast in lean young adults [5]. Similarly, Fallaize (2013) found an increase in satiety, reduced hunger and a lower desire to eat after a breakfast containing eggs with a lower intake of energy at lunch and the evening meal [6]. Ratliff (2010) found that participants ate less energy after an egg compared with a bagel breakfast, and participants ate fewer kilocalories in the whole 24-h period. In contrast, they felt hungrier and less satisfied 3 h after the bagel breakfast [7]. Eggs for breakfast may be helpful during weight loss as shown by Vander Wal (2008) who reported greater weight loss of ~1kg in participants on an energy restricted diet that included eggs for breakfast [11]. There was no effect on weight of including eggs without energy restriction [11].

Possible reasons for the difference seen include the difference in protein intake at breakfast which was 25 g protein from the egg breakfast compared with 11g from the cereal breakfast. Protein is known to be more satiating than other macronutrients [12]. Types of protein may have different effects on appetite as shown by Pal and Ellis [3]. Whey protein reduced appetite and decreased energy intake at a subsequent meal compared with tuna, turkey and egg albumin [3] did not find In contrast a high-fat breakfast resulted in a higher energy intake at lunch compared to a high-protein, low-fat isocaloric breakfast [13]. Energy intake was shown to be similar at lunch following isocaloric breakfasts high in protein, carbohydrate or fat; however, this was a small study of only six participants [14]. A higher protein diet can also help individuals maintain weight loss [15]. In a study in which eggs were eaten at lunch, there was no differential effect on energy intake although while the three lunch meals were isocaloric the differences in protein content were small 21, 16 and 19 g, suggesting that protein may be the more important macronutrient [16]. Fibre is also known to reduce subjective measures of appetite and energy intake, but the effects are small and may depend on the type of fibre used [17]. In the present study, the cereal breakfast meal was higher in fibre, 11 g compared with 7 g, but this did not affect the study outcome.

Limitations of the study were that participants were not asked to record their energy intake after they left the research facility, so it is unclear if total energy intake was reduced for the day. Future studies should include objective measures of food intake for the remainder of the day. Additional studies that would add to the knowledge in this area include conducting longer term studies of eating eggs for breakfast with and without energy restriction.

We conclude that after eating eggs for breakfast, overweight and obese individuals had a lower energy intake at an ad libitum lunch in comparison to eating a cereal breakfast. Both breakfasts contained the same energy. Determining which foods may help overweight and obese individuals manage their food intake is important for diet planning for individuals trying to lose weight.

## Figures and Tables

**Table 1:** BMI—Body Mass Index.

	Men *n* = 16	Women *n* = 34
Age, years	45 ± 22	44 ± 22
Wt, kg	94 ± 16	83 ± 11
BMI kg/m^2^	30 ± 4	31 ± 4

**Table 2:** Energy and macronutrient intake at lunch.

	Mean	SD		Mean	SD	*p*
Energy and macronutrient intake after Egg breakfast			Energy and macronutrient intake after Cereal breakfast			
Total kJ	4518	1593	Total kJ	5284	1814	0.001
Total fat g	11	6	Total fat g	13	7	0.002
Protein g	42	15	Protein g	49	18	0.000
Carbohydrate g	196	70	Carbohydrate g	227	77	0.001
